# Can China’s Environmental Regulations Effectively Reduce Pollution Emissions?

**DOI:** 10.3390/ijerph18094658

**Published:** 2021-04-27

**Authors:** Xi Chen, Zhigang Chen

**Affiliations:** 1 Institute of Central China Development, Wuhan University, Wuhan 430072, China; 2019106320002@whu.edu.cn; 2 Institute of Regional and Urban-Rural Development, Wuhan University, Wuhan 430072, China

**Keywords:** environmental regulation, pollution emissions, green technological progress, nonlinear regression, impulse function

## Abstract

Based on the provincial panel data of China during 2006–2017, this study uses the panel smooth transition (PSTR) model to study the dynamic transformation mechanism of pollution emission under environmental regulation. We focus on technological progress, economic growth, and foreign direct investment (FDI) as threshold variables, and analyses the non-linear effects of environmental regulation on pollution emissions under those threshold variables, attempting to explore the effectiveness of existing environmental regulations. The structure of biased technological progress is based on the slacks-based measure (SBM) and Global-Malmquist–Luenberger index, which is divided into pollution-biased technology progress and clean-biased technology progress. Finally, we use the panel vector auto regressive (PVAR) algorithm to further verify the relationship. The findings are as follows: (1) Environmental regulation has a significant nonlinear effect on pollution emissions, and technological progress is the optimal threshold variable of this study. (2) Under the influence of these three factors, environmental regulation has a substitution effect on pollution discharge, and a stronger substitution effect on emission reduction in areas with advanced technology and high FDI. It also has a lower emission reduction effect in the high-system areas of economic development than in the low-system areas. (3) The PVAR results show that the impact on environmental regulation of technological progress and FDI has gradually turned from positive to negative; the impact of economic growth on environmental regulation has always been positive but is gradually decreasing. This study points out the direction for governments and companies to implement effective environmental regulations.

## 1. Introduction

After nearly 40 years of rapid economic growth through reform and opening up, China has become the world’s second largest economy, and its economic achievements have attracted worldwide attention. However, these successes are mainly due to massive investment in factor resources. In sustainable development, resources and the environment are not only endogenous variables in development, but also rigid constraints on the scale and speed of economic development. Taking gross national product (GDP) as an indicator of economic performance has forced local governments to pursue the increase in economic output and neglect the constraints of investment, which has led some regions to embark on an unsustainable development path of high input and high output. A large amount of resources consumption and low-level technologies have put further pressure on existing environmental conditions. Even in the more developed eastern provinces of China, there are still many companies that produce and supply products with low added value, high energy consumption, and low environmental efficiency. This situation is spreading to central and west China. If this situation cannot be turned around, the profit of Chinese enterprises will not be sustainable and form a path dependency on environmental resources in exchange for economic growth [1]. Although this kind of economic growth model has improved people’s quality of life, the ensuing environmental pollution problems it brings have led China to develop a large amount of environmental regulations in the process of building a resource-saving and environment-friendly society, and will help promote a win–win situation of economic growth and environmental protection) [2].

Faced with the status quo of environmental protection and economic growth, the effective implementation of environmental regulation is an important breakthroughs to solve the contradiction between economic growth and environmental protection [3,4,5]. The representative theories of environmental regulations are as follows. The first involves ‘follow cost’, which is the traditional neo-classic belief that strict environmental regulation will internalize the negative externalities of pollution, indirectly increase the production costs of enterprises, thereby reducing production efficiency and profit. Production scale adjustments, resource allocations, and other behaviour also affect the industrial structure through enterprise entry or exit the market [6]. The second is the ‘pollution refuge hypothesis’, which posits that, in order to avoid restrictions or reduce compliance costs in an open economy, international differences in environmental standards or regulatory levels in terms of trade or investment lead to cross-border transfers of polluting industries [7,8]. This results in adjustments to the national or regional industrial structure. The third theory is the ‘Porter hypothesis’, which posits that strict and appropriate environmental regulations inspire enterprises to change their production processes, guide them to actively seek improved resource utilization efficiency, stimulate technological innovation to reduce environmental compliance costs, produce an innovative compensation effect, and achieve Pareto improvement [9,10].

There are academic disagreements regarding whether greater regulation intensity and stricter environmental standards are required to reduce pollution emissions. Jin [11] indicates that costly environmental regulations eliminate many companies. There is a limit to the number of companies that can be eliminated in a country or region within a certain period of time. Environmental regulations are unsustainable if this limit is exceeded. Xu [12] questions the Porter hypothesis by asserting that it must be based on the ‘proper design of environmental regulation’. Wang et al. [13] consider that strengthening the intensity of environmental regulation might not be conducive for the growth of green total factor productivity (GTFP). Thus, we assume that under different influencing factors, environmental regulation may have a ‘threshold’ for pollution emission. Under the influence of relevant variables in different provinces, environmental regulation has different non-linear effects on pollution emissions. In technology progress path dependence, if technological progress shows more obvious non-green features, the appropriate intervention must be taken to change the direction of technological progress. When the economy is booming, if economic growth brings substantial environmental costs, environmental regulation should be carried out to balance economic development and environmental protection. For the use of foreign capital, if foreign capital inflows contain large numbers of high-pollution and high energy-consuming industries, it is necessary to raise the threshold for foreign capital inflows and eventually put it on a green track.

Environmental issues are a major global challenge facing humanity today [14]. China has always considered sustainable development as an important goal and has implemented many policy measures to reduce emissions, but a consensus conclusion has not been reached on the effectiveness of China’s environmental regulations. This study would not only help to understand the effectiveness of China’s existing environmental regulation policies and efforts but would further enable us to examine how to adjust the factors affecting environmental regulation in order to make emission reductions more effective. In addition, the research in this paper has the potential to provide a corresponding case for a developing country like China.

This study attempts to expand the existing literature from the following aspects. (1) Technology progress is directional. Herein, the DEA method can reveal the hidden or neglected relationship in the environmental system without pre-setting the production function [6], and green technology advancement is calculated. (2) Based on environmental regulation, green technology progress, economic growth, and FDI, the PSTR model is used for empirical analysis. It allows the regression parameters to change gradually and slowly, which can describe the cross-sectional heterogeneity, overcome the endogeneity problem, and avoid the insufficient sample size and group standard arbitrage caused by exogenous grouping better than the threshold model can. (3) Under the effect of different thresholds variables, the PVAR model is used for further binding. We also construct an impulse response function to explore the impact of environmental regulation on pollution emissions and different variables on environmental regulation in order to obtain more effective information.

This paper is structured as follows, with the first part being the introduction. The second part is a literature review on environmental regulation, green technological progress, and FDI on economic development, green efficiency, and environmental protection. The third part is the construction of the model and data description. The fourth part is the empirical analysis, and the fifth part is the conclusion, recommendation and discussion.

## 2. Literature Review

For a long time, the environmental regulation measures of various countries have centred mainly on administrative measures, such as restricting emissions, setting emission standards, and levying pollution taxes, but pollution emission has been allowed to continue through the trading of emission rights. Especially at the macroscopic level, environmental regulation is also dependent on the existing institutional framework for reducing the pollution [15]. Bao et al. [16] propose that environmental legislation cannot significantly inhibit local pollution emissions and can play a significant role only in provinces where environmental law enforcement is strict or local pollution is relatively serious. Zhang and Wei [17] discuss the nonlinear relationship between environmental regulation and carbon emissions, and point out that when the intensity of environmental regulation changes from weak to strong, its role should be changed from "green paradox" effect to the ‘reverse emission reduction’ effect; Yu and Gao [18] analysed the impact of environmental regulation on environmental pollution from the perspective of the informal economy. The higher is the intensity of environmental regulation, the greater is the negative impact of the invisible economy on the Chinese environment.

In addition, most scholars consider a government’s environmental regulation when analysing environmental protection and emission reduction, and also take into account the use of advanced technology and clean technology [19], gradually involving the direction of technological progress. Technological progress can improve the efficiency of resource use and reduce resource consumption while increasing economic output, especially the biased technological progress [20]. Economic growth and the advancement of new-type industrialization need to be supported by technological progress, technological progress will also promote environmental protection [21]. Up to now, research on internationally biased technology progress has been based on the results of Acemoglu [22]. Most research on biased technology in China is based on Dai and Xu [23]. Rational environmental regulation can change the direction of technological progress, which would help Chinese industry to drive green technological progress [24]. Song and Wang [25] based on the data envelopment analysis (DEA) method, measure technological progress by dividing it into environmental-biased and pollution-biased, and find that environmental-biased technological progress can improve environmental quality, while an ageing population would encourage the use of pollution-biased technologies.

In related research on green development, Zhao [26] believes that increased of environmental regulation would slow regional economic growth in China, which has become an insurmountable challenge in the process of industrialisation. Environmental regulation has restrained regional economic development by increasing the production cost of enterprises [27], and it has improved the quality of regional economic development by stimulating technological progress. However, environmental regulation can have different effects in different regions. The population, green industry, and environmental regulation in areas with high green development levels for industry have a greater role than the low green development areas in promoting industrial green development [28].

In terms of the relationship between environmental regulations and FDI, a more relaxed degree of environmental regulations attracts FDI inflows from polluting industries [29], and stronger environmental regulations reduce the negative impact of FDI on host country productivity, and environmental regulations play an important role in screening inward FDI [30]. Environmental regulations play a positive role in enhancing the quality of incoming FDI and the quality development of the Chinese economy [31].

The existing research focuses on the impact of environmental regulation on production efficiency, technological innovation, industrial optimisation, FDI, and openness, which provides a rich theoretical basis for this research. The degree of environmental regulation affects the inflow of FDI, the degree of economic development, and local technological progress, plays an important role in macroeconomic operations. In contrast to the existing literature, we try to consider green technological progress, GDP and FDI as the threshold variables of environmental regulations on pollution emissions, and explore whether environmental regulations can achieve emission reductions as expected under the combined influence of these three factors, which is a complement and extension to the existing literature.

## 3. Models and Data

### 3.1. Models

#### 3.1.1. Panel Smooth Transition (PSTR) Model

This study used the PSTR proposed by González et al. [32]. The model is a further extension of the panel threshold regression, which can be used to characterize the cross-sectional heterogeneity of panel threshold data. The model set-up is as follows:

(1) $$y_{it}=\mu_{it}+b_{1}{}^{\prime }x_{it}+\sum_{k=l}^{l}b_{k}{}^{\prime }x_{it}g\left(q_{it}{}^{\left(k\right)};\gamma ,c\right)+\varepsilon_{it}(1)$$

where *y_it_* indicates the pollution emission intensity of region *t* in the *i*-th year, *x_it_* indicates the environmental regulation, *μ_it_* indicates the fixed-effect value of the section unit, *b_1_’* and *b_k_’* indicate the parameter to be estimated; and *ε_it_* is the random disturbance term. $g_{k}\left(q_{it}{}^{\left(k\right)};\gamma ,c\right)$ is a continuous change function between 0 and 1; the observable value *q_it_* is the conversion variable; *γ* is the smoothing parameter, which determines the speed of the conversion; and *c* is a positional parameter that occurred for the conversion. $g_{k}\left(q_{it}{}^{\left(k\right)};\gamma ,c\right)$ usually takes the following logical functional form:

(2) $$g\left(q_{it};\gamma ,c\right)={\left(1+exp\left(-\gamma \overset{m}{\underset{j=1}{\prod }}\left(q_{it}-c_{j}\right)\right)\right)}^{-1}\;c_{1}\leq c_{2}\leq \ldots \leq c_{m},\gamma >0$$

The PSTR model starts from the information implicit in the data themselves and is grouped by estimation. When the transfer function $g_{k}\left(q_{it}{}^{\left(k\right)};\gamma ,c\right)=0$, the model corresponds to a low system; when the transfer function $g\left(q_{it};\gamma ,c\right)=1$, the model corresponds to a high system. The conversion function values are smoothly converted between 0 and 1, allowing the model to transition smoothly between the two different systems.

If γ→+∞, the PSTR model is transformed into a panel threshold regression model for system transformation. This is because if $q_{it}>c$, $\underset{\gamma \to +\infty }{\lim}-\gamma \left(q_{it}-c\right)=-\infty$, that is $\underset{\gamma \to +\infty }{\lim}g_{1}\left(q_{it};\gamma ,c\right)=1$ and conversely, if $q_{it}<c$, $\underset{\gamma \to +\infty }{\lim}g_{1}\left(q_{it};\gamma ,c\right)=0$, that is,. At this point, the PSTR model has two limit states: $y_{it}=\mu_{it}+b_{1}{}^{\prime }x_{it}+\varepsilon_{it}$ and $y_{it}=\mu_{it}+{\left(b_{1}+b_{2}\right)}^{\prime }x_{it}$. Therefore, the panel transition model can be regarded as a special case of the PSTR model; if $q_{it}=c$ or γ→0, $g_{1}\left(q_{it};\gamma ,c\right)=0.5$ the PSTR model degenerates into a fixed-effect model.

#### 3.1.2. Panel Vector Auto Regressive (PVAR) Model

This study used the PVAR model for further analysis of the relationship between pollution emission intensity and environmental regulation, and the impact response between variables. The model uses a multi-equation approach to analyse and predict interconnected multivariate systems and to explain the impact of various shocks on economic variables. This model has less theoretical requirements and needs to determine only the variables to be studied and the maximum order of lag. It is not necessary to determine whether the variables are endogenous or exogenous, which overcomes the defects pointed out by Arrow et al. [33], who criticize the environmental Kuznets curve (EKC) assumption that income is an exogenous variable. The PVAR model not only integrates the advantages of panel data and vector auto regression, but also reduces the restrictive requirements of the vector autoregressive method for time-series length, but can also capture the influence of sample unit individual differences on model parameters [34].

The PVAR model in this study uses the variables, such as pollution emission intensity, environmental regulation, green technology progress, economic growth, and FDI. By considering the heterogeneous characteristics of unobservable individuals, a panel impulse response function is established, which can isolate the impact of each variable on other variables and analyze its influence on other variables. Considering technological progress, economic growth, and FDI as threshold variables, the study constructed environmental regulations as explanatory variables; we first constructed the impulse response function of environmental regulation and pollutant emission intensity, and then the impulse response function of the other three variables and environment regulation.

### 3.2. Data

#### 3.2.1. Description of Each Variables

When measuring the global-Luenberger-Index of the inefficient change of technology, the desirable output is regional GDP, the undesirable outputs are wastewater and gas emissions of each city and province, and the input index is fixed asset investment, the number of employees in the labor force, and total energy consumption. When calculating the PSTR model and the PVAR model, the following indicators are selected. The variables and the symbols in the following regression are shown in the Table 1.

#### 3.2.2. Calculation of Key Variables

(1) Calculation of pollution

In general, two methods are used to measure the intensity of pollution emissions: first, pollution reduction costs and expenditure indicators, and second, a variety of pollutants and total pollution after the determination of industrial pollution intensity. This study referred to Lu and Wei [35] and Li and Tao [36] to calculate pollution emission intensity, selecting industrial wastewater discharge and industrial waste gas emissions from 30 provinces between 2006 and 2017. The quantity related data were linearly standardised, equal weighted, and averaged to calculate the pollution intensity of each province and city. This study calculates the pollution emission value of the pollutant output value of each province and city, that is, where is the emissions of the *j*-th pollutant in the *i*-th province and city and is the industrial added value of each province and city. In this study, it is linearly normalized, equalized and averaged to obtain.

(2) Calculation of er

China’s 10th and 11th Five-Year Plans both mention a 10% reduction in China’s Sulphur dioxide (SO_2_) emissions [37]. SO_2_ removal rate is one of the main goals of sustainable development in China [38]. SO_2_ removal rate is a widely used environmental regulation variable, both in terms of government objectives and in terms of society-wide impacts. Therefore, we also use SO_2_ removal rate as a proxy for environmental regulation. SO_2_ removal rates prior to 2011 were taken from the China Environmental Statistics Yearbook. After 2011, the statistical calibre has changed, SO_2_ removal rates is calculated by the method of (SO_2_ generation − SO_2_ emission)/SO_2_ generation.

(3) Calculation of techg

**Figure 1:**
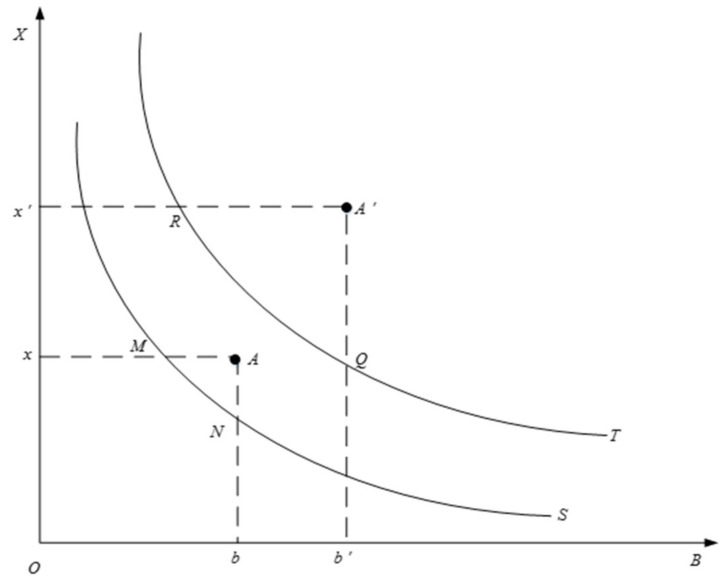
Plan view of the change of front surface of DMU (decision-making units) production in three-dimensional space. *X* is an input element, such as capital and labour. *B* is wastewater and exhaust gas emissions, that is, undesired output. In s period, *x* is the input element of Euclidean centre *A*, *b* is its undesired output, and *S* is the production frontier. In t period, *x*′ is the input of the Euclidean centre *A*′, *b*′ is its undesirable output, and *T* is the production frontier. *R*, *Q* and *M*, *N* are the intersections of *A* and *A*′ with the production fronts *T* and *S* respectively.

According to Acemoglu et al.’s [39] biased technical progress definitions, and simulation of the production boundary with the DEA algorithm by Song et al. [40], we calculate the two-sector model containing clean and polluting production technologies under the open economic conditions established by Jing and Zhuang [24]. Thus, we can obtain the technological progress needed for this study. This model allows the two techniques to progress at different speeds, thereby portraying the bias of technological advancement. The DEA measures the relative technical rate of change and requires multiple decision-making units (DMU) to construct the production frontier. The direction of technology change is estimated by the offset of the boundary position, but the direction of the progress of single DMU technology cannot be judged. At the same time, the DEA method must also determine DMU benchmarks to reflect the technological changes of all DMUs. Based on these considerations, we use the slacks-based measure (SBM) method, which is more common in measurement technology, to simulate the offset process of the production front, as shown in Figure 1. In this figure, *X* is an input element, such as capital and labour. *B* is wastewater and exhaust gas emissions, that is, undesired output. The total energy consumption axis *E* passes through point *O* and is perpendicular to the principal plane. In s period, *x* is the input element of Euclidean centre *A*, *b* is its undesired output, and *S* is the production frontier. In *t* period, *x*′ is the input of the Euclidean centre *A*′, *b*′ is its undesirable output, and *T* is the production frontier. *R,Q* and *M,N* are the intersections of *A* and *A*′ with the production fronts *T* and *S* respectively.

The results show that the position of the production frontier is shifted, and the position of Euclidean centre *A* in the production envelope is also changing. Therefore, when measuring the progress of technology changes, we must consider position deviation *A* on the production front. If, under the same undesired output, inputs and outputs increase at the end stage more than at the beginning, or if the output increases under the same input conditions and the undesirable output decreases, the production frontier moves toward the *X* direction, which is called clean technology progress, that is, $\rho_{X}=\frac{Nb}{Ab}/\frac{Qb^{\prime }}{A^{\prime }b^{\prime }}$. Conversely, if the DMU exhibits a further increase in undesirable output, that is, the production frontier is biased toward *B*, this shows pollution technology progress, that is, $\rho_{B}=\frac{Mx}{Ax}/\frac{Rx^{\prime }}{A^{\prime }x^{\prime }}$. In the production process, if there are more resources and energy consumption of inputs, undesirable output increases at this time, which leads the initial technology to show more obvious non-green characteristics. If energy consumption is lower, but use of existing capital and labour are higher, the undesired output is reduced, resulting in the initial technology showing more obvious green features. Technological advances reinforce this trait and show continued green or non-green bias. Therefore, the green technology progress of this study is defined as *Techg = ρ_x/_ρ_B_.* When, *Techg >* 1 that is, technological progress is located in the production frontier or is superior to it, there is clean technology progress; when *Techg <* 1, technological progress fails to reach the production frontier for polluting technology progress [41].

This study selected the additive Luenberger index proposed by Fukuyama and Weber [42] to measure green technology progress. The Luenberger index is based on relaxation of the directional distance SBM function [23]. The SBM-based global directional distance function and the global Luenberger index were constructed based on the global Malmquist-Luenberger index proposed by Oh [43]. The global production frontier was constructed after detecting the production technology over the entire time period, which avoids the ‘technical regression’ and the unsolvable linear programming phenomenon that can occur with the mixed directional distance function. In conjunction with cyclic accumulation, the global Luenberger index can be used not only to analyse short-term changes in technology, but also to observe long-term trends. On this basis, the global Luenberger index, which calculates the technical inefficiency change, can be expressed as ${\mathrm{GL}}_{t}^{t+1}=S^{G,k^{\prime }}\left(t+1\right)-S^{G,k^{\prime }}\left(t\right)$.

(4) Calculation of GDP

We adopted green GDP to represent China’s total output when considering environmental constraints. Green GDP represents the level of economic development in each region, for which we used the year 2000 as the base period for deflation. We then used GDP minus the depreciation of fixed assets and the cost of environmental protection to obtain green GDP. The cost of environmental protection includes environmental protection inputs, waste gas, and wastewater multiplied by unit value.

(5) Calculation of FDI

The ratio of the actual use of FDI to gross national product (FDI/GDP). The actual use of foreign investment is converted to US dollars at the end of each year.The current research objective does not include an empirical analysis of Tibet, Hong Kong, Macao, or Taiwan because of the lack of data availability. This study selects the relevant data from 2005 to 2017 to calculate the technical progress data from 2006 to 2017. The other data are from 2006 to 2017. Relevant data were selected using the China Statistical Yearbook, China Demographic Yearbook, China Environment Yearbook. Descriptive statistics of the data are shown in Table 2.

## 4. Empirical Analysis

In this study, the global-Luenberger-index was calculated using Maxdea software, and regression analysis was performed using MATLAB R2016a.

Since the panel data model contains both time and space dimensions, in order to avoid the pseudo-regression caused by the unit root, the unit root test is performed based on possible cross-sectional correlation (Table 3). The test results show that the *p* values of all variables strongly reject the null hypothesis that there is a panel unit root.

### 4.1. PSTR Model Analysis

The PSTR model allows the influence of environmental regulation on the intensity of pollution emission to be smoothed with different influencing factors, that is, the influence of environmental regulation on the intensity of pollution emission is not mutated at a certain breakpoint, or it is strictly linear relationship with each influencing factor. The influencing factors investigated in this paper are green technology progress, GDP and FDI. And the model effectively overcomes the problem of group regression, traditional threshold regression and so on, which cannot accurately depict the smooth conversion of variable coefficients and improves the accuracy of the results. The model is mainly based on the regression of fixed effects. Before the regression, the Hausman test was performed on the models of three different threshold variables. The test results strongly reject the null hypothesis and suggest that a fixed-effect model should be adopted. Then, the green technology progress, GDP, and FDI are used as threshold variables, and it is verified whether the non-linear effect of environmental regulation on pollution emissions under the influence of these variables is established. Table 4 illustrates the test results for the different threshold variables.

The test results show that the regression coefficients of each variable of the three models pass the significance test, indicating that environmental regulation has significant non-linear effects on pollution emissions in various regions. The heterogeneity of regions leads to significant differences in the emission reduction effect of environmental regulation among different provinces. The existence of conversion variables leads to the division of the elasticity coefficient of environmental regulation to pollution emission into different systems, and the elasticity coefficient is smoothly transformed between systems. In addition, Colletaz and Hurlin [44] mention that the optimal threshold variable is statistically large. According to this standard, green technology advancement is the most important factor in the non-linear relationship between environmental regulation and pollution emissions. Table 5 shows the regression result.

(1) Green technology progress and environmental regulation of emission reduction effect. The estimated results from the Model (1) indicate that the model has a positional parameter of 0.3123. The model is divided into two systems, with the observed value in the high system being 162, and the sample observation value in the low system 198. With the change of conversion variable *techg_it_*, the reduction elasticity of environmental regulation is smoothly converted between the high and low systems, and the rate of change is 7.6888. When *techg_it_* < 0.3123, the model is located in the low system, and environmental regulation increases pollution emissions. Environmental regulations increase pollution emissions by 0.0792 units per unit, but not significantly. When *techg_it_* > 0.312, the model is in a high system, and the elasticity coefficient of environmental regulation to pollutant emissions is −0.2581 (the elasticity coefficient is *b_0_* + *b_1_*). An increase of 1 unit of environmental regulation reduces pollution emissions by 0.2581 units. Taking the average of technological progress in each province and city for 12 years, there are 14 provinces (i.e., Beijing, Tianjin, Liaoning, Shanghai, Jiangsu, Zhejiang, Anhui, Fujian, Shandong, Guangdong, Hainan, Chongqing, Ningxia, and Qinghai) are located in the high system. These areas have made technological progress of a cleaner type through lower energy consumption and waste emissions. Most other regions are eastern provinces and municipalities, with superior infrastructure conditions and factors, and their technological progress also favours the clean type. The remaining 16 provinces and cities are located in the low system, which shows that most provinces need to change the mode of economic development, reduce the level of energy consumption per unit, and improve environmental regulation, thereby reducing the intensity of pollution emissions.
(2) Emission reduction effect of GDP and environmental regulation. The estimated results from Model (2) indicate that there is a positional parameter of 0.1931 and the model is divided into two systems. In this study, we take the natural logarithm of GDP and all observations are above the position parameters. With the change of conversion variable *gdp_it_*, the reduction elasticity of environmental regulation is smoothly converted between the high and low systems, and the rate of change is 30.1989. When *gdp_it_* < 0.1931, the model is located in the low system, and elasticity coefficient of environmental regulation to pollution emissions is 0.1594; that is, when the environmental regulation increases by 1 unit, pollution emissions reduce by 0.1594 units. When *gdp_it_* > 0.1931, the model is located in the high system, and the elasticity coefficient of environmental regulation to pollution emissions is 0.1454; that is, when the environmental regulation increases by 1 unit, pollution emissions reduce by 0.1454 units. It is noteworthy that when the model is located in the low system, the impact of environmental regulation on pollution emission is greater than in the high system, the total GDP of the low system is smaller, the cost of environmental regulation may exceed the scope of some enterprises, and the effect of implementation may be more obvious. However, the high system area, namely, the existing observation value, no longer blindly pursues economic growth, allows the enterprise to pay a certain environmental cost to carry on production, and implements the local environment regulation policy. Although the pursuit of high-intensity environmental regulation can effectively promote emissions reduction, it can increase sewage costs by a large amount for the normal operation of enterprises.
(3) The emission reduction effect of FDI and environmental regulation. The estimated results from Model (3) indicate that there is a positional parameter of 0.0962, and the model is divided into two systems. The observed value in the high system is 354, and the observed value in the low system is 6. With the change of conversion variable *FDI_it_*, the reduction elasticity of environmental regulation is smoothly converted between the high and low systems, and the rate of change is 88.8741. When *FDI_it_* < 0.0962, the model is located in the low system, and the elasticity coefficient of environmental regulation to pollution emissions is −0.0003; that is, when environmental regulation increases by 1 unit, pollution emissions reduce by 0.0003 units, but it is not significant. When *FDI_it_* > 0.096, the model is in a high system, and the elasticity coefficient of environmental regulation to pollution emissions is −0.0353; that is, when the environmental regulation increases by 1 unit, pollution emissions decrease by 0.0353 units. Under the influence of *FDI_it_*, no matter what kind of system in which the model is located, increasing environmental regulation reduces pollution emissions. In recent years, China has developed a higher degree of openness to the outside world, attracting much high-quality foreign investment, improving the technology for reducing emissions, and preventing the inflow of high energy-consuming industries and backward industries to a large extent, so that the production of high-system environmental regulations is more obvious than that of low-system regulations. Even if some provinces and cities are located in the low-system stage, although the emission reduction effect is not significant, the local environmental regulation still shows the emission reduction effect.

### 4.2. Impulse Response Analysis

To further verify the relationship between environmental regulation, green technology progress, economic growth, and pollution emissions, this study established a PVAR model with variable lag term to study the change relationship of each variable. The panel co-integration test was performed using the Kao test and Pedroni test before the regression. The results show that both tests strongly reject the null hypothesis that there is no co-integration relationship.

**Figure 2:**
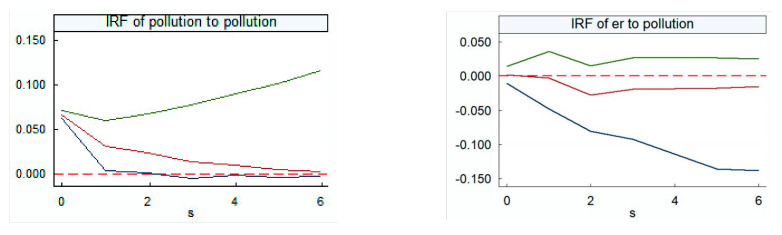
Environmental regulation and pollution emission impulse response diagram. On the **left** is an impulse response plot of the pollution emissions on itself, and on the **right** is an impulse response plot of the environmental regulation on pollution emissions. IRF—impulse response function.

**Figure 3:**
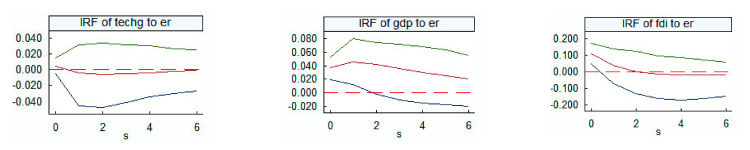
Impulse response graphs for the relationship of each variable to environmental regulation. Note: On the **left** is an impulse response plot of the techg on er, the **middle** one is an impulse response plot of the gdp to er, and the **right** one is an impulse response plot of the fdi to er. IRF—impulse response function.

Considering the role of threshold variables, this study first considered the impulse response relationship between environmental regulation and pollution emissions, and then further explored the impulse response relationship of each threshold variable to environmental regulation. The results are shown in Figure 2 and Figure 3. According to the minimum principle of the AIC and SIC tests, the model choice is the lag 3 period for analysis of the influence of environmental regulation and pollutant emissions, and the lag 1 period for analysis of the influence of other variables on environmental regulation. Considering the sample capacity of this study, the research period is set to six periods.

The model passes the stability test, showing that the PVAR system should be established. Since this model does not focus on specific economic theories, it is of little significance for analysis of coefficients. Therefore, after establishing the PVAR model, this study uses the impulse response function to explain the relationship between green technology progress, environmental regulation, economic growth, and pollution emissions from a dynamic perspective. The horizontal axis of Figure 2 shows the period of the impulse response analysis as six periods. The vertical axis represents the response intensity of the interpreted variable to the explanatory variable, the middle solid line represents the trajectory of the impulse response function, and the outer two curves represent the confidence interval.

The positive influence of pollution emission intensity itself occupies the dominant position, and although its effect shows a decreasing trend over time, its effect is the largest of the two variables. Until the end of the study, the impact of pollution emissions on their own tends to be zero, meaning that in the late stage of the study, the increase in pollution emissions in the previous period reduced the positive contribution to pollution emissions in the current period, indicating that the current emission reduction measures to a large extent can reduce pollution emissions. The impact of environmental regulation on pollution emission intensity is positive before the first period, and then shows a downward trend. After the second period, the convergence begins to rise, but it still has a negative impact. This means that the increase in environmental regulation in the first period raises pollution emissions. This may be because the early implementation of environmental regulations increased the cost of sewage, so that enterprises were encouraged to transfer environmental pollution of the official economy into the shadow economy. In real life, most enterprises discharge pollutants by paying bribes or using other rent-seeking methods without paying sewage charges or purchasing pollutants. The pollutants of these enterprises have not even reached discharge standards, thereby still increasing pollution emissions. After entering the Eleventh Five-Year Plan period, China’s central government officially established a resource-saving, environment-friendly society as a strategic priority for the medium- and long-term planning of national economic and social development, and a role for environmental regulation began to emerge. Improved environmental regulation began to reduce pollution emissions. Its role peaked in the second phase, and then gradually converged, but environmental regulation still has a negative effect on pollution emissions.

Green technological progress in the first period gives the environmental regulation a positive impact after an initial decline, and then a negative impact. In the second period, the negative effect peaks, but remains negative and tends toward 0. This may be because for a lot of provinces and cities at the beginning of pollution-type technology progress, pollution-based technology progress on energy efficiency is low, cannot effectively reduce emissions, and promotes environmental regulatory intensity after the second phase gradually increases in order to reduce pollutant emissions. However, up to the end of the study, there is a negative impact, that is, the speed of environmental regulation decreases slowly under the influence of technological progress. In the later stage of the study, the technological progress of some provinces gradually changes from pollution to clean type. The use of clean technology progress increases the popularity of clean energy or clean technologies in the production process and reduces the cost of cleaner production. The improvement of green technology reduces the degree of environmental regulation but can further promote the realization of emission reduction based on suitable environmental regulation measures. The impact of green technological progress on pollution emissions is more stable, indicating that technological progress has a certain time lag on environmental regulation, and it is a continuous accumulation process.

After the first period, when economic growth has a positive impact on environmental regulation, it begins to decline, but remains positive. This means that economic growth is conducive to increasing the degree of environmental regulation at the beginning of the period, but thereafter, the role begins to decrease although it remains positive up to the end of the study. In the later stage of the study, economic growth slows the increase of environmental regulation, and the effect of environmental regulation on pollution emissions needs to be of the right degree, that is, in this stage, China begins to pay attention to the effectiveness of environmental regulation rather than increase the intensity of environmental regulation. The above research results show that environmental regulation has a lower emission reduction coefficient when GDP is high than when it is low. This finding is consistent with the abovementioned results, indicating that China has begun to implement rational environmental regulation.

FDI has a positive impact on environmental regulation at the beginning of the study, and the increase in FDI contributes to the increase in environmental regulation. After the second period, FDI has a negative impact on environmental regulation, and the increase in FDI reduces environmental regulations, although this impact is slow. This means that FDI may be accompanied by more backward industries through industrial transfers from developed countries at the beginning of the period. In order to help China from becoming a ‘pollution paradise’, the inflow of FDI at the beginning of the period strengthens the degree of environmental regulation to a certain extent. However, in the later period, FDI may be accompanied by more high-quality foreign trade and foreign capital. The degree of environmental regulation decreases with the inflow of this FDI in order to attract more advanced technologies, but this trend tends to converge.

It was found that green technology progress and FDI have negative impacts on environmental regulation in the later period. However, in both these high-system areas, increased environmental regulation results in higher emission reduction elasticity, which means that green technology progress and FDI replace the role of some environmental regulation. However, a better combination of the two with environmental regulation could effectively reduce emissions, which highlights the importance of formulating reasonable environmental regulation policy in China. The impact of GDP on environmental regulation is positive throughout the study period, but the development of the economy in the later period slows the improvement of environmental regulation, which means that environmental regulation in the GDP growth process no longer increases blindly. Moreover, the environmental regulation in the high-system area has less elasticity, which indicates that China has gradually attached importance to environmental regulation measures based on local conditions and has found other suitable ways to reduce emissions.

## 5. Discussion

This study has the following policy implications. In the context of the relatively unfriendly environmental impact of China’s economic activities, it is very difficult to reduce the consumption of environmental resources by relying on existing economic operations. It is necessary to formulate a reasonable environmental regulation policy. In addition, the effects of environmental regulation are gradually emerging. Under the so-called ‘new normal’ economic situation of more moderate economic growth, the non-linearity of environmental regulation and pollution emission has been supported by many scholars. Green technology progress, economic growth, and FDI have different impacts on environmental regulation. The emission-reduction effects of environmental regulation can be adjusted rationally by improving these three factors.

Based on the results, this study makes the following recommendations:

(1) The Chinese government should strongly support R&D for clean technology, such as encouraging leading new energy and new technology industries to vigorously strengthen their technology R&D, subsidise corresponding R&D funding, attract the inflow of technicians and actively promote the transformation of clean technology from research to application and further to large-scale production.
(2) China’s government should accelerate the establishment of low-carbon and green growth models in some of backward regions, avoid repeating the "pollute first, treat later" model, raise the threshold of environmental regulations in backward regions to prevent pollution transfer, and establish a mechanism for the elimination of winners and losers among enterprises to prevent unqualified enterprises from entering the market.
(3) Relevant government departments should implement regional policies for the introduction of FDI. The Chinese government should identify and control the quality of FDI through environmental regulation tools. It is necessary to provide policy support and tax incentives for clean FDI, impose appropriate fines for heavily polluting FDI, and gradually establish production incentives for green investment.
(4) Relevant government departments should develop and implement different types of appropriately designed environmental regulatory instruments to achieve better emission reductions. Local governments should actively adjust their regulatory efforts according to economic development and the current environmental situation in order to leapfrog the turning point of some of the macro variables affecting emissions reduction. Each region should systematically try out progressive environmental regulation policies based on actual needs, economic characteristics and stage of development, especially in some inland underdeveloped regions, and set up a fund to support green development and provide financial support for the green transformation of industries in backward regions.

## 6. Conclusions

In recent years, China’s development has exposed increasingly serious environmental problems. This study analysed the non-linear relationship between environmental regulation and pollutant emission intensity under the influence of green technological progress, economic growth, and FDI. Among them, technological progress is based on the biased technical progress analysis framework, and the global-Luenberger-index based on the SBM model was constructed to measure China’s green technological progress. With green technological progress, GDP, and FDI as the threshold variables and pollution emission intensity as the explanatory variable, the non-linear relationship between current environmental regulation and pollution emission intensity in China was discussed, and the relationship between the variables was verified using the impulse response function in the PVAR model.

The results show that the environmental regulation is significantly non-linearly affected by the impact of green technological progress, economic growth, and FDI on pollution emissions.

(1) Under the effect of low-system technological progress, the increase of environmental regulation brings about no significant pollution emission increase, but it can significantly reduce pollution emissions under the effect of a high system of technological progress. Green technological progress at the beginning of the pollution-biased period corresponds to a large amount of pollutant emissions, so the environmental regulation increases. Enterprises facing higher sewage costs inspire their economic people characteristics through rent-seeking or free-rider behaviour to seek profits, not only to promote the level of current environmental regulation, but to increase their own pollution emissions. Clean technological progress improves the efficiency of technology and energy use in the production process, reducing the damage of production on the natural. Furthermore, clean technology progress has been developing and replacing pollution technology progress, effectively recycling resources, reducing pollution emissions of unit output, and lowering environmental regulation in the latter stage, but the research period has a slight rebound. The emission reduction effect of environmental regulation is more significant under the effect of high-system technological progress.
(2) Under low-system economic growth, the implementation of environmental regulation can significantly reduce emissions, but it should be within the appropriate scope, while high-system economic growth reduces the emission reduction effect of environmental regulation. When the economy develops to a certain extent, in addition to environmental regulation, the role of other emission reduction measures is more obvious. For most of the study period, economic growth has led to a decrease in the speed of environmental regulation, which indicates that China began to focus on adopting appropriate environmental regulation measures, rather than high-intensity environmental regulation measures, to reduce the sewage cost of enterprises under high environmental regulation intensity, and to maintain the emission reduction effect of environmental regulation.
(3) Under low-system FDI, environmental regulation has no significant emission reduction effect on pollution emissions, but under high-system FDI, there is a more obvious inflow of environmental regulations faced by a large number of foreign direct investors, which has a higher effect on emission reduction. FDI increases the degree of environmental regulation at the beginning of the study. From the second period, FDI increases reduce environmental regulations, but this change is slow. This may be due to the low quality of FDI flowing into China at the beginning of the period, accompanied by the transfer of backward industries. Although the degree of environmental regulation is high, the emission reduction effect of foreign capital is not high. In the later research period, the inflow of advanced technology reduces the intensity of environmental regulation, but greatly increases the efficiency of environmental regulation.

This study not only considers the emission reduction effect of environmental regulations, but also introduces whether environmental regulations can be effective in reducing emissions under the influence of some macroeconomic variables such as green technological progress, GDP and FDI. In addition, the study also explores the impact of these three macroeconomic variables on environmental regulation. The effectiveness of environmental regulation to reduce emissions in China is conditional on the influence of macro variables, and this paper provides suggestions for a more precise solution to achieve emissions reductions. In addition, the use of the PSTR model distinguishes itself from traditional threshold models by allowing for slow changes in macro variables and by effectively dealing with endogeneity, making it a more realistic model. However, this study also has certain limitations in that we only validate three macro variables and cannot put other macro variables to the test one by one. In addition, the environmental regulation in this study only uses the more influential and widely used sulphur dioxide removal rate. The real strength of the government’s environmental regulation is difficult to measure, and if it can be measured in the future, we can use it as a next step to better explore how policies can be implemented to better achieve emission reductions.

## Figures and Tables

**Table 1:** Variables and explanations.

Variable Category	Variables	Symbols	Unit	Data Sources
Dependent variable	pollution emission intensity	pollution	-	China Statistical Yearbook
Independent variable	Environmental Regulation	er	%	China Environmental Statistics Yearbook
Threshold variables	Green technology progress	Techg	-	China Statistical Yearbook
	Green Gross Domestic Product	GDP	Natural logarithm	
	Foreign direct investment dependence	FDI	%	

**Table 2:** Descriptive statistics.

Variables	Observation	Mean	Standard Error	Min	Max
pollution	360	0.249	0.187	0.008	1.000
er	360	0.492	0.243	0.000	0.997
techg	360	0.374	0.203	0.152	1.173
gdp	360	8.351	1.039	4.456	10.354
fdi	360	2.392	1.948	0.028	11.809

Note: pollution—pollution emission intensity; er—environmental regulation; techg—green technology progress; gdp—green gross domestic product; fdi—Foreign direct investment dependence.

**Table 3:** Unit root test.

	Im-Pesaran-Skin Test	Augmented Dickey-Fuller Test	Phillips-Perron Test
pollution	−2.5827 (0.0049)	6.3653 (0.0000)	6.3653 (0.0000)
er	−4.0395 (0.0000)	6.5238 (0.0000)	6.5238 (0.0000)
gdp	−4.0543 (0.0000)	8.4371 (0.0000)	7.4747 (0.0000)
fdi	−2.6471 (0.0041)	10.6568 (0.0000)	3.9952 (0.0000)
techg	−2.4480 (0.0072)	7.5099 (0.0000)	12.8959 (0.0000)

Note: pollution—pollution emission intensity; er—environmental regulation; techg—green technol-ogy progress; gdp—green gross domestic product; fdi—Foreign direct investment dependence.

**Table 4:** Non-linear test results.

Threshold Variables	*H_0_:γ = 0*	*H_1_:γ = 1*	*H_0_:γ = 0*	*H_1_:γ = 1*
	Lagrange Mutiplicator (LM)	Fisher Lagrange Mutiplicator (LMF)	Lagrange Mutiplicator (LM)	Fisher Lagrange Mutiplicator (LMF)
techg	18.834 ***	18.162 ***	0.015	0.013
	(0.000)	(0.00)	(−0.903)	(−0.908)
gdp	9.811 ***	9.217 ***	0.002	0.002
	(−0.002)	(−0.003)	(−0.966)	(−0.967)
fdi	4.666 **	4.320 **	0.149	0.136
	(−0.031)	(−0.038)	(−0.699)	(−0.716)

Note: techg—green technology progress; gdp—green gross domestic product; fdi—Foreign direct investment dependence. *** *p* < 0.01, ** *p* < 0.05.

**Table 5:** Model regression result.

	(1) techg	(2) gdp	(3) fdi
Slope coefficient *b_0_*	0.0792	−0.1594 ***	−0.0003
	(−1.2578)	(−12.3715)	(−0.0218)
Slope coefficient *b_1_*	−0.3373 ***	0.0140 ***	−0.0350 ***
	(−4.1304)	(−4.2129)	(−2.6883)
Position parameter *c*	0.3123	0.1931	0.0962
Conversion speed parameter *r*	7.6888	30.1989	88.8741
Akaike information criterion (AIC)	−3.600	−4.202	−3.6888
Bayesian Information Criterion (BIC)	−3.556	−4.159	−3.645

Note: techg—green technology progress; gdp—green gross domestic product; fdi—Foreign direct investment dependence. *** *p* < 0.01.

## Data Availability

No new data were created or analyzed in this study. Data sharing is not applicable to this article.
